# Riding the wave into wellbeing: A qualitative evaluation of surf therapy for individuals living with acquired brain injury

**DOI:** 10.1371/journal.pone.0266388

**Published:** 2022-04-07

**Authors:** Katie Gibbs, Lowri Wilkie, Jack Jarman, Abigail Barker-Smith, Andrew H. Kemp, Zoe Fisher

**Affiliations:** 1 School of Psychology, Faculty of Medicine, Health & Life Science, Swansea University, Swansea, United Kingdom; 2 Regional Neuropsychology and Community Brain Injury Service, Morriston Hospital, Swansea, United Kingdom; 3 Health and Wellbeing Academy, Faculty of Medicine, Health & Life Science, Swansea University, Swansea, United Kingdom; Monash University, AUSTRALIA

## Abstract

Nature has long demonstrated the capacity to facilitate wellbeing. Interventions involving the natural environment such as surf therapy, are increasingly being used to facilitate aspects of wellbeing in clinical populations. However, explorations of how nature-based interventions such as surf therapy may be used to promote wellbeing in the context of neurorehabilitation are missing from the peer-reviewed literature. Here we characterize the experience of a five-week surfing intervention involving fifteen adults living with the psycho-social and cognitive sequelae of acquired brain injury. Insights were analysed using reflexive thematic analysis, which highlighted the importance of seven overarching themes, including: 1) Connection to Nature, 2) Facilitating Trust and Safety, 3) Managing and Accepting Difficult Emotions, 4) Facilitating Positive Emotion, Meaning and Purpose, 5) Building Community through Social Connection, and 6) Positive Change. Barriers and opportunities (theme 7) were also identified as components on which clinical services may be improved. We present a theoretical model for the benefits of surf therapy in people living with acquired brain injury (ABI) based on these themes and reflections on findings from the wider literature. Findings emphasise the importance of leveraging community partnerships to augment the holistic model of neurorehabilitation and potential implications for service redesign are discussed, focusing on recent developments in wellbeing science.

## Introduction

An estimated 1.3 million people live with the effects of brain injury at a cost to the UK economy of £15 billion per annum, a figure that is equivalent to 10% of the annual NHS budget [1]. For the individual, Acquired Brain Injury (ABI) can have wide-ranging and often pervasive effects across physical, emotional, cognitive, and social domains [2]. The psychological impact of ABI typically comprises profound psychological distress, with prevalence rates for depression post-injury ranging from 27%-64% [3–5]. Neuro-behavioural problems can impact on existing relationships, contributing to poor family functioning and loss of social networks [6]. Over time, poor psychological wellbeing and isolation ensues [7, 8] reinforcing persistent barriers to social integration even after 10+ years post-injury [9]. Despite the pervasive impacts of ABI [10–12], developments in existential positive psychology have emphasised tremendous capacity for wellbeing despite suffering [13, 14].

The ‘Holistic Neurorehabilitation Model’ [15, 16] has been shown to be more effective than traditional neurorehabilitation approaches which focus on the reduction of deficits and distress [17, 18]. The holistic approach considers the dynamic relationship between a person and their environment, and respects the reciprocal relationships that exist between psychological, social, cognitive, and physical domains of wellbeing following injury [16, 19–21]. However, even within Holistic models of Neurorehabilitation there is often still a tendency for neurorehabilitation efforts to focus on reducing deficits and distress, rather than also on facilitation of health and wellbeing [22, 23]. This is despite evidence that health and wellbeing is not simply the absence of impairment [6]. For example, the dominant model used to treat psychological difficulties post ABI has been Cognitive Behavioral Therapy (CBT) which aims to reframe unhelpful negative thoughts in order to reduce negative affect and psychological distress. In contrast, approaches which aim to also increase positive traits, emotions and experiences within the ABI population have shown promise despite being in their infancy [24]. For example, group and one-to-one positive psychotherapy has been reported to increase happiness [25] and reduce symptoms of anxiety [26]. Our own qualitative analysis of a positive psychotherapy intervention for people living with ABI reported upon themes of ‘Empowerment’, ‘Social Opportunity’, ‘Coping’, ‘Cultivation of Positive Emotion’, ‘Consolidation of skills’ and ‘Barriers to Efficacy’ [27].

We further argue that wellbeing interventions have been developed with a focus on isolated components. For instance, positive psychological interventions [28, 29] are often distinct from the promotion of other determinants of individual wellbeing, which include positive health behaviours [30, 31], social relationships and community cohesion [32, 33], nature connectedness [34, 35] and the ability to initiate and sustain positive behaviour change [36] (see [37] for a comprehensive review). Accordingly, our work draws from a much more expansive theoretical basis than the field of positive psychology and we argue that models of neurorehabilitation can be enhanced further by drawing on broader theories of wellbeing and advances in wellbeing science [22, 23]. We recently defined wellbeing [38] from a biopsychosocial ecological perspective, emphasising connectedness to the self, others and nature, which may reflect a basic psychological need, supported by vagal function, a psychophysiological resource for connection. We also emphasise that wellbeing is impacted by socio-contextual factors that lie beyond the control of the individual. These determinants of wellbeing and their interrelatedness are captured within our life-course theoretical GENIAL framework [23, 37–39], the acronym for which captures the relationships that occur between Genomics and its interaction with the Environment through to health outcomes, highlighting a major regulatory role for the vagus Nerve over social Interaction and Allostatic regulation, subsequently leading to premature mortality or Longevity. This biopsychosocial model imposes an interpretative framework on an otherwise heterogeneous and disconnected body of literature, integrating findings from wellbeing science and drawing on the available peer-reviewed literature to highlight pathways through which wellbeing can be realised. Here, we emphasise core domains of wellbeing at different levels of scale: including the individual domain (including a balanced mind and a healthy body); the community domain (social connectedness), and the environmental domain (connection with nature). We also highlight the role of socio-contextual factors that either facilitate or restrict the experience of wellbeing. Our theory is explicitly linked to a broader context and is consistent with an abductive or explanatory approach to theory generation [40] that addresses many of the criticisms and controversies that have dominated wellbeing science and moves towards a transdisciplinary model of wellbeing. Accordingly, we argue that integrating these insights from wellbeing science provides opportunities for models of neurorehabilitation to promote wellbeing beyond a focus on happiness or reducing negative emotions.

Our nature based Surfability intervention, described herein, is an example of expanding the holistic model of neurorehabilitation by focusing on determinants of wellbeing that are often neglected. The Attention Restoration Theory emphasises the restorative effects of spending time in nature on attention and concentration [41, 42] which may be particularly useful for people with ABI. Other scholars argue that exposure to unthreatening natural environments help to reduce physiological arousal following stress [43, 44] and increase resilience [45], in line with stress reduction theory [46, 47]. The potential for nature to facilitate resilience may be particularly important in the context of brain injury populations [48]. Nature can meaningfully reduce psychological and physiological markers of stress and replace them with feelings of refreshment and vigour in as little as 10–20 minutes [49]. Contact with nature has also been shown to improve cognitive functioning [50, 51] and facilitate the experience of psychological flow [52] and there is now a growing body of evidence for the wellbeing benefits associated with engagement in water-based activities [53]. A systematic review of 35 studies concluded that exposure to outdoor blue spaces is positively associated with higher levels of physical activity, better mental health, and improved wellbeing within the general population [54]. Psychological processes including empowerment and respite have been proposed as potential mechanisms through which surfing may facilitate wellbeing [55].

Surf therapy provides a context for the experience of multiple determinants of wellbeing. The potential for exercise to serve as an immediate psychological reward for the continuation of health behaviours that support self-management is particularly important in the context of individuals with brain injury [56, 57] (see also [58]). Connection with nature may also have benefits over and above benefits associated with exercise [59–61]. Here we explore whether our Surfability intervention–built on strong theoretical foundations [22, 23, 37–39]–can facilitate wellbeing in a group of service users living with ABI. The benefits of surfing in the context of this unique population remain–until now–unexplored. Accordingly, the aim of this study is to characterise the experiences of a surfing intervention in individuals living with the residual effects of brain injury, and to reflect on potential mechanisms through which reported improvements in wellbeing may function in a conceptual model.

## Methods

### Participants

The service evaluation was carried out in a community neurorehabilitation service, set in a general hospital setting in South Wales, United Kingdom. This service encompasses the Traumatic Brain Injury Service (which primarily works with individuals who have experienced mild or moderate and severe brain injury), the Vocational Stroke service (which accepts referrals from stroke patients who wish to return to employment) and the General Neuropsychology Service (which accepts referrals for people living with Acquired Brain Injury). All services provide clinical and neuropsychological support, with standard care across services centred around the Holistic Model of Neurorehabilitation. This involves working with service users to identify goals that they wish to work on as part of their rehabilitation. This may include brain injury education, vocational rehabilitation, and interventions to help service users compensate for difficulties, adjust to their circumstances and new identity, and work towards rebuilding a different meaningful life when returning to their ‘old’ life is not possible. Various therapies are offered to help patients achieve their rehabilitation goals. For example, the Traumatic Brain Injury Service is composed of a multidisciplinary team including speech and language therapists, occupational therapists, music therapists and clinical psychologists/neuropsychologists. In keeping with the Holistic Model of rehabilitation, almost all service users will engage in a combination of individual and group therapies.

As part of their ongoing treatment and rehabilitation, service users were invited to attend one of three Surfability interventions delivered over a three-year period (2018–2020). These interventions were offered during the latter months of each year (July-October) in accordance with the optimum sea temperature and seasonal weather conditions. These environmental variables impacted upon the numbers of participants able to access this intervention within the focus of the current study. Sample size was also further restricted by the COVID-19 pandemic, which resulted in the cessation of in person group-based neurorehabilitation services during the first year of nationwide lockdown. As such, a total of 25 participants were purposely invited to attend the intervention and 18 accepted the invitation. Reasons for not accepting the invitation included other commitments and travel difficulties. All 18 participants attending one of the three Surfability interventions over the three-year period were invited to provide qualitative feedback about their experience of the intervention. Of these 18 participants, three were lost at interview because they could not be contacted to make an appointment or because they failed to attend the interview appointment that had been made for them. Accordingly, 15 participants consented to take part in the service evaluation. Table 1 shows demographic data for the 15 participants who provided qualitative information about their experience of the Surfability intervention.

**Table 1:** Participant characteristics.

Age	Mean = 42.4; Standard Deviation 12.88; Age range (29–69 years); Median = 38
Sex	Male = 10; Female = 5
Type of Acquired Brain Injury	Severe Traumatic Brain Injury *n* = 6; Moderate Traumatic Brain Injury *n* = 3; Moderate-Severe Traumatic Brain Injury *n* = 1; Mild-Moderate Traumatic Brain Injury *n* = 1; Mild Acquired Brain Injury *n* = 1; Pontine Cavernoma Bleed to the brain *n* = 1; Subarachnoid Haemorrhage secondary to a ruptured right middle cerebral artery aneurysm *n* = 1; Multiple Sclerosis diagnosed in 2006 *n* = 1
Time Since Injury	Mean = 2 years and 9 months; Standard deviation = 3.07; Range = 6 months– 12 years; Median = 2 years
Employment Status	Employed *n* = 3; Employed but on sickness leave; *n* = 2; Medically retired *n* = 3; Unemployed *n* = 7.

In accordance with the eligibility criteria for the service, all participants had a confirmed diagnosis of ABI, were aged 18 years or older, lived in the community and catchment area of the health board, and were able to actively engage in neurorehabilitation, as determined by treating clinicians. Participants were only invited if they were able to provide informed consent to participate. All participants experienced cognitive difficulties, particularly in relation to memory, processing, and executive function. Risk assessments were carried out by clinicians, and all staff were made aware of participant needs and individual requirements. Individuals deemed not medically fit to partake in physical activity were excluded from the intervention, with uncontrolled epilepsy comprising the only reason the service has had to exclude potential participants on the basis of physical health grounds. Physical difficulties which needed to be managed for almost all participants during the intervention included fatigue, dizziness, and balance difficulties. Participant needs were accommodated due to adaptations made by Surfability to increase accessibility.

### Ethical considerations

Evaluations of service user experiences associated with the delivery of interventions in the healthcare sector are excluded from ethical review in the United Kingdom (GAfREC §2.3.12). This exemption was confirmed by the Research and Development Officer in Swansea Bay University Health Board. Service evaluations are characterised by minimal risk and therefore fall outside the remit of research ethics committees in the United Kingdom. There was no experimental component or randomisation, nor was the intervention withheld for any reason from eligible participants. Patient care did not deviate from the typical care provided by the service from which the data was generated. All participants were invited to participate in focus groups and interviews and all participants agreeing to participate provided consent and were provided with the option to leave at any point prior to recordings being made. All participants valued the opportunity to provide feedback on our intervention to help inform future service development. Recordings were transcribed without identifiable information, and the individual analysing the transcripts was not involved in data collection. Our findings are interpreted and discussed in the context of the peer-reviewed literature and healthcare improvement.

### Design

A qualitative evaluation (QE) design was employed to gather detailed accounts of service user experiences of the Surfability intervention, consistent with national requirements for evaluating services and patient experience. This work is in keeping with a participatory and context-sensitive approach, in line with our previously published work [22, 27].

### Intervention

A five-week Surfability therapy intervention was delivered in a collaborative partnership between a community neurorehabilitation service (CNS) in a major hospital located in South Wales, and a local community-based third sector surfing organisation. Surfability UK (https://surfabilityukcic.org) is a Community Interest Company that provides surfing experiences for individuals with additional needs due to disability, illness, injury or learning difficulties. The aim of Surfability is to help people with impairment to push past the boundaries of what they thought possible and engage in safe, group-based physical activity in a natural outdoors environment. Surfability UK is located at Caswell Bay on the Gower Peninsula of South Wales. This coastal setting is accessible to individuals with restricted mobility and is renowned for its aesthetically pleasing environment, facilitating psychological and physical connectedness to nature, laying strong foundations for building wellbeing in people living with ABI.

The intervention consisted of weekly two-hour sessions which took place in groups of no more than 5 participants. Groups were led by three qualified surf instructors employed on the Surfability project, in addition to two members of therapy staff from the CNS, plus volunteers. Each group consisted of sufficient staff to provide one-to-one support to participants, with high-needs individuals (including those with physical disabilities). Participants had sufficient time (usually about 30-minutes) before the session to meet staff and other group members and to change into their wetsuits before the surfing intervention commenced. Adapted wetsuits with extra zips and heated vests were available for participants who struggled to put on their wetsuits, thus preventing the cold from being a barrier to inclusion. Surfing activities lasted for 1 hour and 30 minutes, commencing at 10am and finishing at 11.30am.

At the start of the first session, appropriate surf boards and buoyancy aids were distributed amongst participants by the Surfability team. Surfability has a range of different sized surf boards which vary in length, width, flexibility, firmness, and level of adaptation to suit those with physical limitations. For instance, if a person’s physical difficulties meant they were unable to use a traditional board even with support, then Surfability has a large surfboard with a seat attached to it. Use of this seated board would involve a coach paddling out and catching waves so the seated participant could still experience the thrill of being on a wave and in the ocean. Another coach would be waiting at the end of the wave to provide support if necessary. This meant that physical disability was not a barrier to participating. Although there was no one in the reported sample with severe mobility problems, all participants in the current evaluation required adaptations to the size and firmness of their boards to compensate for difficulties with balance and to increase stability. For participants who had difficulties walking to the sea due to mobility issues, beach-access wheelchairs and walking frames were available to support them.

Once boards were selected, the lead instructor provided verbal instructions regarding Surfing skills and outlined suitable positions to take while on the board. A thorough physical demonstration of surfing techniques was provided; including how to lay down, kneel and stand up on the board. Verbal health and safety instructions were provided, and participants were encouraged to raise their hand and call for assistance if or when support was needed. Surfing activities typically began with staff members supporting participants to lay down (where possible) on the board while in the sea. Staff members would help participants to ride the waves of the sea, by pushing them onto the waves. This process was repeated so that participants could practice the skills necessary to balance on the board while focussing on the movement of the sea and the waves. This would continue until participants felt that they no longer required the support from staff members and had learnt to successfully ride the waves for themselves. Participants were able to take short breaks whenever they felt it was appropriate and would often sit with peers and observe the group activities during breaks.

Informal goal setting and progress monitoring comprised an integral component of the intervention. Clinical team members at the CNS worked with participants to identify initial informal goals prior to engaging within the intervention. These goals typically reflected individual reasons for taking part and often reflected psycho-social outcomes and opportunities, including socialising with new people, and learning about the experiences of other brain injury survivors. During each week, goals tailored to the needs and requirements of each participant were revisited and participants were encouraged to reflect on their progress and outline actions that they wanted to achieve for that day. Sessions were carefully designed to bring clients out of their comfort zone and help them: make room for difficult experiences/feelings; focus on positive experiences such as the feeling of being in the water; bringing awareness to the present moment, and the experience of belonging to a group. Meanwhile, informal goal setting was included to facilitate achievement and the broadening of skills.

### Data collection

All interviews were conducted by one of two Assistant Psychologists (AP) (both female) with postgraduate training in psychology. Neither AP had established relationships with participants but had relevant clinical experience in working with individuals with ABI. Twelve interviews were conducted face-to-face in a hospital setting and three were conducted via telephone. During face-to-face interviews, two participants were accompanied by a member of their support circle (i.e., parent or support worker) as requested, and clinical staff were in the building (for governance reasons) but not in the interview room. Before the interviews began, participants were made aware of the purpose of the discussion and were informed that their anonymized data would be used for evaluative purposes. Each interview followed a similar pattern and discussions were framed around experiential gains, points for improvement and salient aspects of the course which may have impacted upon individual wellbeing (see S1 File). Discussions were semi-structured in nature and utilised open-ended questions, with follow-up queries developed iteratively over the course of the discussions. This approach facilitated flexibility and responsiveness, consistent with best practice in qualitative research.

All interviews were recorded using a Dictaphone and participants were assigned a numerical identifier during transcription to protect their identity. Audio data of interviews totalled 6 h and 52 min. Interviews were on average 27 minutes and 45 seconds long, ranging from 10 minutes and 41 seconds to 45 minutes and 30 seconds in duration (SD = 10.70). Audio files were transcribed orthographically (incorporating utterances, hesitations, false-starts and repetitions) and utilized grammatical correctness to ensure the true essence of the data was captured. Interviews were transcribed verbatim except for the names of participants, staff names and locations, which were omitted to ensure confidentiality. Anonymised transcripts were stored on a secure shared drive within the health board.

### Data analysis

A reflexive approach to Thematic Analysis was employed to explore participants’ subjective experiences of the Surfing intervention and synthesize participant responses into meaningful accounts [62, 63]. This approach embraces researcher subjectivity and allows for a researcher to draw upon their unique knowledge, skills, and experiences to generate insight into the lived experiences of participants. Using our GENIAL framework as a lens through which participant’s experiences could be understood and contextualised, all coding was conducted by the first author (KG), a Ph.D. candidate with clinical experience and relevant knowledge about factors which may facilitate and/or restrict opportunities for wellbeing in individuals with chronic conditions. Coding was completed according to a critical realist epistemological perspective [64], an approach which emphasizes the role of the individual in making their own subjective meaning of human experience, while also acknowledging that this experience is historically and culturally situated and driven by a broader social context. As such, this perspective is one that supports the use of theory in generating understanding.

Data analysis was supported by computer-assisted qualitative data analysis software (ATLAS.TI) and guided by the six-phase process described by Braun and Clarke [62]. Following extensive familiarisation with and re-reading of the data, these phases outline a recursive process of coding participant transcripts to capture provisionally interesting features *within* the dataset and developing candidate themes and subthemes that capture the essence of shared meaning *across* the data set. Candidate themes and subthemes were reviewed and continuously refined while a descriptive narrative was created to contextualise the outcome of the analysis. Meaningful quotes which exemplified the core features of each theme were extracted from the data set and used to tell a coherent story regarding participants’ experiences of the Surfability intervention. During this reflexive process, members of the research team (ZF and AK) contributed their insights and expertise to help shape the analytic output, providing directives to facilitate a rich and clear reading of the data. While this feedback supported the first author (KG) to further refine and develop the analytic output, the results were ultimately shaped by the first author’s individual nuances and expertise.

## Results and discussion

All participants who provided feedback on the Surfability intervention reported psychosocial difficulties secondary to brain injury, including cognitive difficulties, negative affect, job loss and relationship breakdown. Many felt that the experience of sustaining an ABI had changed their lives for the worse such that they struggled to come to terms with their diagnosis. This background information provides important context for the seven themes characterised below.

### Overarching themes

**Fig 1:**
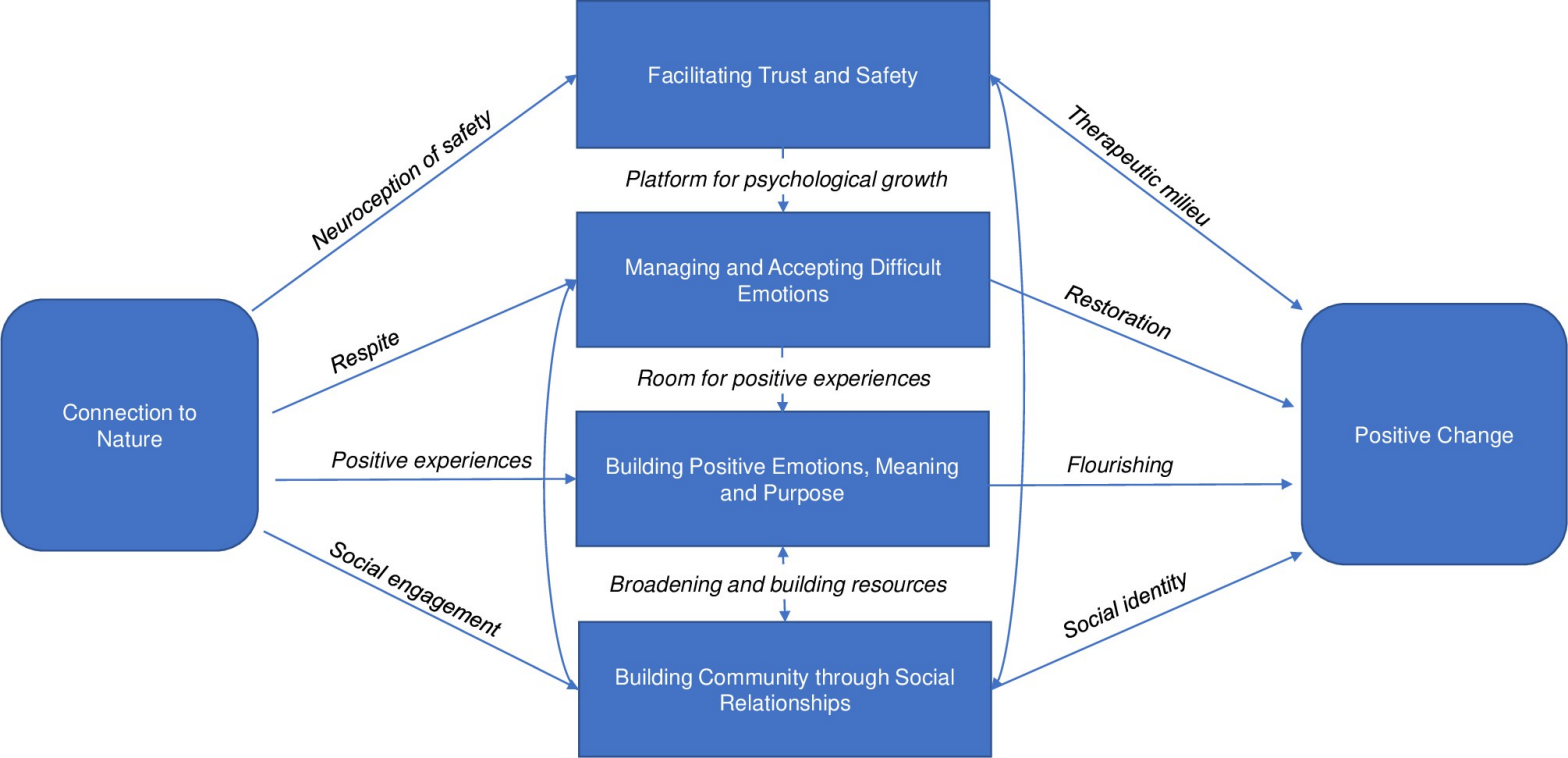
A proposed conceptual model for the benefits of surf therapy in people living with acquired brain injury, illustrating potential relationships between overarching themes and potential underlying mechanisms. Figure from Kemp & Fisher (2022) (CC BY 4.0).

Seven overarching themes were generated, including: 1) Connection to Nature, 2) Facilitating Trust and Safety, 3) Managing and Accepting Difficult Emotions; 4) Facilitating Positive Emotion, Meaning and Purpose, 5) Building Community through Social Connection; 6) Positive Change, and 7) Barriers and Opportunities. We now discuss these themes in more detail and place them within the context of the broader literature, reflecting on potential underpinning mechanisms by which these themes may facilitate wellbeing. A summary of how the overarching themes and potential mechanisms might relate to each other is provided in Fig 1.

### Theme one: Connection to nature

The nature-based component of Surfability provided participants with the opportunity to interact with their natural environment, contributing to experiences of empowerment, a sense of relaxation, revitalization and refreshment, and an appreciation of beauty regarding the coastal location in which surfing took place.

*“The scenery we have got in South Wales is some of the best in the world and seeing that—being able to be grateful to see that, it definitely has lifted me.” (Participant Seven)*

The Nature Restoration Theory argues that nature may have restorative effects on our attention and concentration such that spending time in natural, yet aesthetically pleasing environments takes us away from the effortful attention-demanding tasks associated with the urban world [41, 42]. Participants described feeling beneficial effects of being in the open water that are consistent with theories of attention restoration.

*“Surfing especially—it’s physical, but not too overly demanding, you know? It’s the sensations of the water, the smells, it’s really good for the senses.” (Participant Ten)*

Exposure to unthreatening natural environments may also reduce physiological arousal following stress [47], such that immersion in a natural and social ecology took participants away from everyday stressors, if only for a short while.

*“I love the water, it’s the feeling of safeness again, just being able to feel, you know. Feel the waves, the elements.” (Participant Thirteen)*

Some participants reported feeling more connected to nature following engagement in the intervention, with one participant expressing a preference for outdoor surfing as opposed to other natural outdoor environments [34].

*“It’s [the Ocean] just so calming. I don’t know. That’s something that’s changed. I always enjoyed swimming and the relaxingnness of the water, but I just feel as if I’m more connected to water than trees.” (Participant Thirteen)*

Nature connectedness is a key prerequisite for health outcomes that affects the extent of psychological benefits obtained through spending time in the natural environment [65] and is considered one path to flourishing in life [34, 37, 56]. We argue that connecting with nature facilitated a variety of benefits reported by participants, with such benefits reflected in the following themes. These include the facilitation of trust and safety, managing and accepting difficult emotions, evoking positive emotions and a sense of meaning and purpose, as well as promoting a sense of community, all of which will support capacity for positive change (Fig 1).

### Theme two: Facilitating trust and safety

This theme captures how the creation of a safe and supportive environment empowered participants to push past their self-doubt and anxieties and to focus upon re-building their confidence and capacity for self-management. Participants reported feeling safe and secure in the group and indicated that the presence of a qualified network of skilled professionals and surf instructors provided them with the opportunity to try something new without fear, harm, or ridicule. Here is an exemplar quotation from Participant Five:

*“I thought this was an environment where it didn’t matter if I made a tit of myself, it didn’t matter. I would feel safer if I was with someone who was proficient in their field, you know? They were able to teach this. I did it because it felt a safe environment for me to be able to learn something that I had never done before. And I think that is quite precious, that the opportunity was given.” (Participant Five)*

The impact of trauma affects the nervous system, altering cues of risk and safety, a process described as neuroception, which can lead to the perception of threat even when the environment is safe [66, 67]. This mismatch results in physiological and behavioural states that support defensive and avoidant strategies, compromising the ability to detect and to express positive social cues, leading to social withdrawal [68]. We suggest that connecting with nature facilitated feelings of safety, an experience that was supported by Surfability staff and clinicians. For example, one participant emphasized how the Surfability team saw past their diagnosis and treated them as capable individuals with the means to achieve.

*“Everybody who works in the team, they don’t patronise you, they don’t see you as ‘Oh you have this, or you’ve got that condition.’ They just see you. ‘Right OK, you want to learn to surf, let’s see you get on that board.’ You know? And I think that is the best place to be. I think that it’s just that they see the person in front of them, and not the condition, and I think that is really important”. (Participant Three)*

Our findings align with the Holistic Model of Neurorehabilitation which outlines the importance of the ‘therapeutic milieu’ in determining positive neuro-rehabilitation outcomes. The therapeutic milieu refers to the sense of safety, trust, and co-operation which if cultivated maximises the adjustment processes, facilitates social participation and promotes positive change [17] (Fig 1). The facilitation of trust and safety can lay a platform for psychological growth, including the capacity for better managing and accepting difficult emotions, a key overarching theme that we discuss next.

### Theme three: Managing and accepting difficult emotions

Almost all participants who engaged in the Surfability intervention reported experiencing psychological distress, anxiety and/or depression prior to participating in the group. Increasing evidence suggests that surfing in an ocean environment can facilitate feelings of respite and enhance wellbeing in individuals with lasting psycho-social difficulties [55, 69] by necessitating a focus on body and mind in the present moment [70]. In line with these findings, participants who attended the Surfability intervention reported feelings of stress reduction, indicating that surfing had interrupted bouts of rumination arising from relationship breakdown and challenging personal events.

*“It was just like a break from my mind if I’m honest. Out in the sea I literally didn’t think of anything else that was happening in my life.” (Participant Eleven)*

The experience of surfing also facilitated acceptance of difficult emotions. Here, participants indicated that the unpredictable and uncontrollable nature of the water taught them that they could not always control the occurrence of unpleasant events and feelings. This included, for example, the cold weather, the size of the waves and getting hit by the board or falling into the water (at least before practice enabled them to develop their skills). As such, participants learnt that unpleasant feelings (beyond their control at that point in time) were an integral feature of the human experience: insights which were reinforced by clinicians who tried to make these links explicit.

*“I think that it sort of proved to me that I don’t have to know everything—what’s going to happen—all the time. Because I can’t control it and I think that’s what it taught me, in the water, I can’t control that. Whatever is going to happen, whether it hits me in the face, or I fall off the board, it’s going to happen. And I can’t control that. So just sort of go with it.” (Participant Three)*

Some participants reported that these insights helped them to better manage difficult emotions and experiences beyond the parameters of the Surfability intervention, indicating that they were able to generalise experiences in surfing to other aspects of their lives.

*“I can now step back and say the majority of the time ‘Come on, you know you can’t control this, why are you freaking out?’ or ’Why are you crying, why are you panicking?’ You know?” (Participant Three)*

Acceptance of one’s mental experience has been linked to better adjustment and functioning in individuals with chronic conditions [71–73] and improved psychological health within the general population [74]. This may be because individuals who are better able to accept difficult thoughts and emotions experience reduced negative emotions in response to stressors [74]; facilitating opportunities for improved psychological wellbeing, including meaning and purpose, the theme we turn our attention to next.

### Theme four: Positive emotion, meaning and purpose

Surfability provided opportunities for positive psychological experience, with many participants describing a ‘boost’ or a ‘buzz’ suggestive of feelings of revitalisation, refreshment, and vigour. Engagement in the group made brain injury survivors ‘feel alive’ (i.e., Participant Thirteen), with one participant exclaiming that it gave them ‘a new lease of life’ (Participant Three). Some participants highlighted the impacts that physical exercise had on their mood and perceptions, consistent with evidence indicating that (even minimal levels of) aerobic exercise can have a positive impact on emotional balance in those living with brain injury [75].

*“It’s as if, sort of like a dirty duvet cover–you put a dirty duvet cover in the washing machine, and you come out and you feel sort of refreshed then. That’s how I feel when I go.” (Participant Thirteen)*

Surfability provided brain injury survivors with opportunities to engage in and master their surroundings [76, 77] and pursue clearly identified and meaningful goals with autonomy and control. Some participants reported experiencing a flow-like state during the session, such that they felt immersed in the experience and were so focussed on overcoming the challenges inherent to surfing that they lost track of time (Participant Four). Positive psychological experiences were facilitated and reinforced through goal setting exercises, supported by clinicians working with patients during the Surfability sessions. Feelings of achievement cultivated joy, gratitude, and hopefulness for the future, with one participant expressing how lucky they felt to be alive (Participant Six).

*“You’ve got the board, you’ve got the waves, you’ve got the sea, and nobody is sitting next door to you with dual controls, and that’s a heck of an achievement.” (Participant Six)*

Surfability also facilitated aspects of eudaimonia, with group members reporting a new sense of meaning and purpose in life. Specifically, engagement in the group contributed to a sense of direction and the perception that life was worthwhile.

*“To have something that I enjoyed that made me want to get up, get dressed, to prepare for that was amazing, I think that’s really beneficial. ‘Cause it encourages a good lifestyle, healthy lifestyle. It encourages you to do anything because you can still do stuff and you have got a valid reason for being alive.” (Participant Five)*

Research indicates that engagement and meaning are more strongly related to the experience of wellbeing than that of pleasure [78], again highlighting the potential for surfing interventions to optimise wellbeing in participants with chronic conditions. This is particularly important, given that opportunities for meaning and achievement following ABI are lacking due to barriers to employment [79, 80], with only 20% of participants in the present sample being able to return to work. Moreover, positive experiences may broaden mindsets in ways that build social resources over time [81], facilitating community integration in persons with ABI [23], the theme we turn to next.

### Theme five: Building community through social connection

Surfability provided an opportunity for brain injury survivors who had previously reported feeling isolated to experience greater social connections and form meaningful social ties with individuals from diverse backgrounds, reinforcing the role of social connectedness in surf-related positive health and wellbeing outcomes [55, 69]. Being in a group with similar others facilitated a sense of belonging and identity through shared life experiences [82], with one person describing their peers as a ‘family’ connected by a mutual understanding of experience (Participant One). Here, participants provided emotional support to their peers through expressions of encouragement and reassurance [83]. These emotionally supportive interactions fostered feelings of belonging in participants, helping them to feel accepted and valued despite personal difficulties. Meanwhile, seeing similar others succeed by sustained effort motivated participants to ‘go back to the shore and get straight back on again’ (i.e., Participant Five), potentially enhancing levels of self-efficacy through vicarious learning and experience [84].

*“What was good about it was you could see other people—not that I am trying to put them down—but they were falling off as well as I was falling off. Yet, when each individual person did it, it was a group where we all applauded then, which was like bonding. You weren’t trying to put somebody down—we were hoping that they could get up!” (Participant Six)*

Relating to other survivors through ‘sameness’ helped to combat social isolation and build a sense of community [85]; facilitating social cohesion and cultivating teamwork. Participants provided informational support to their peers [83], sharing coping mechanisms, knowledge, and values with the aim of supporting others to live well with injury:

*“You know you’re sitting in that chair, and you think ‘What is going to happen to me?’ And you’re just ticking over in your own mind. But now, seeing these people here and even people with head injuries, you can talk to them, and they are giving their experience. And maybe I can give my experience and that will help them.” (Participant Six)*

Sharing within this network contributed to the building of trust and learning of new skills [86, 87]. Feelings of trust and belongingness are basic psychological needs which are important for renewal of self-identity after brain injury [88]. Meanwhile, the sharing of resources between individuals from diverse backgrounds united by social and/or cultural cleavages also provide the grounds for bridging social capital, increasing acquisition of knowledge and social resources which facilitate adaptability and successful self-management [37]. Moreover, the experience of mutual identification, shared understanding, and sense of belonging in this context may foster positive psychological and physical health outcomes in individuals with chronic conditions [89], and improve quality of life [90] and adjustment [91] in individuals with acquired brain injury.

### Theme six: Positive change

The Surfability intervention provided a context for sustained positive change both within and beyond the parameters of the Surfability project itself, nourishing the belief that despite ‘being a bit broken in some places’ (Participant Four), participants were capable of achieving and experiencing wellbeing. This enabled brain injury survivors to continue building a better version of themselves, such that participants reported positive changes across psychological, cognitive, physical, and behavioural domains.

Firstly, making room for difficult thoughts enabled participants to (re)connect with themselves in terms of their values, hobbies and identity and focus on re-building a new version of themselves, shifting their focus from their ailments and towards their capabilities. These changes contributed to the development of self-efficacy, an aspect of cognitive self-appraisal that helps to reduce discrepancy between achievements and expectations [92].

*“It made me feel like ‘Oh my god, you can still do stuff! Oh, my goodness me, you just sat up on a surfboard, amazing, and you’re 47, you know, cracking!” (Participant Five)*

Some participants reported that Surfability had increased or improved their awareness, attributing these changes to the fluid and dynamic movements of the tide, wind, and sea. For example, one participant spoke about how they became more aware and observant of their surroundings during daily activities such as cooking, doing laundry or sitting as a passenger in a car. The activity of surfing thereby facilitated more mindful perceptions in participants, facilitating a deeper awareness of oneself and one’s surroundings in other day-to-day activities. This suggests that mindful experiences facilitated over the course of the intervention generalised to other experiences within participants’ daily lives, perhaps characteristic increases in trait mindfulness and contributing to a less distressed disposition [93].

*“I do like being outdoors, you know the gardening outside but how can I say it—the garden stays still, doesn’t it? Surfability doesn’t stay still, and you are achieving or getting your mind to work on what’s going on around you. You’ve got to be aware of what’s going on around you because Surfability is a thing that’s moving, and you are not really in control of it. Although you are on a surfboard, you are trying to guide it, it’s keeping you aware then and you’ve got to be aware. You are looking around to see if there is anyone else in your way that you don’t want to go into people, so it does bring awareness to you.” (Participant Six)*

Participants also reported that attending Surfability helped them to engage in positive health behaviours outside of the group by providing a more pleasurable form of exercise than gym-based workouts.

*“I have dropped out of the gym so many times because it’s just been too much. But, with Surfability, I can’t explain it—it’s an exercise but it’s a pleasure and it doesn’t wear me out as much as the gym does.” (Participant One)*

Participants reported improved fitness, coordination, and balance, with some participants reporting increased uptake of nature-based physical activities following the intervention.

*“What Surfability has given me is that it has helped me with my coordination, my fitness, getting me out the house, yeah, getting wacked by mother nature really.” (Participant Two)*

In this regard, Surfability re-ignited pre-existing affiliations with the outdoors for many participants. For others, it provided the opportunity to build such an affiliation, with several participants expressing that they have wanted to spend more time outdoors and explore new places since engaging in this nature-based intervention.

*“I feel like I want to be a bit more outdoorsy, I don’t want to be sort of hidden away anymore.” (Participant Three)*

These outcomes support the notion that exercise in the natural environment may have a greater effect on physical and mental wellbeing than exercising indoors [59]. In addition to improving physical health outcomes in brain injury survivors [94, 95], participation in exercise also plays a critical role in emotional balance [75] and improving one’s capacity for self-management [96]. Finally, Surfability provided the opportunity and context for new skill development and repetition of that skill–a key component of successful behaviour change [23]. The development of positive health behaviours and sustained exercise regimes are particularly important for brain injury survivors, given risk of sedentary behaviours [97].

### Theme seven: Barriers and opportunities

This theme captures the essence of barriers experienced by brain injury survivors that may impede upon their ability to benefit from therapy in any context. Firstly, some participants reported difficulties in accessing holistic healthcare interventions altogether, with one participant indicating that their needs had not been met by more traditional services with a focus on reducing illbeing. Of those who could access more holistic therapies, consensus dictated that the activities to which brain injury survivors were referred to previously were not suited to their needs, interests, or values.

*"I think that I have been through so many things in the hospital where I have either I have not been believed or just given the pill” (Participant Three)*

This feedback highlights a need for greater attention to be directed towards identifying a range of programmes to suit the needs of a heterogeneous client group, as well as the importance of involving participants in the design and delivery of healthcare [98]. Moreover, it emphasizes the utility of building strategic and creative partnerships between the healthcare sector and community organisations, such as the Community Interest Company (Surfability) described herein, in order to provide innovative healthcare. Research has shown marked social inequalities regarding access to and use of blue spaces, with financial constraints restricting engagement in blue space activities in individuals from lower-socioeconomic backgrounds [99]. The surfing intervention described herein is an exemplar of how socio-structural barriers may be circumvented by partnering with community providers; a collaborative approach which lays important foundations for a more sustainable healthcare sector. Meanwhile, individual-specific barriers that were identified in this study may be used to improve the delivery of wellbeing therapies in the context of brain injury. Specifically, clinicians should be aware of participant fatigue, which was reported by six participants, assumptions regarding abilities (Participant Five) and older participants comparing themselves unfavourably with their younger peers regarding progress made (Participant Thirteen). These factors may have restricted the efficacy of the Surfability intervention itself and reflect common barriers to neurorehabilitation. In this regard, one potential limitation of the study concerns the lack of cultural diversity within the sample, in that different cultural values differentially influence determinants of wellbeing [37]. For example, in individualistic cultures, wellbeing has been shown to be more strongly associated with self-esteem and sense of personal achievement. In contrast, collectivist cultures place more value on social harmony, with wellbeing being more strongly associated with avoiding social conflict and interpersonal goals [100]. However, the sample in the present study is reflective of the typical population accessing community neurorehabilitation services within the general hospital setting in question.

### Clinical implications

Our research shows that developing collaborative interventions with community partners may add value by facilitating access to community resources and allowing for neurorehabilitation interventions to make use of the emerging benefits associated with the natural environment [61, 101, 102] in addition to those associated with positive health behaviours [30, 103, 104]. These rich insights denoting participants’ experiences of surf therapy may be complemented by quantitative and/or longitudinal research to investigate the sustainability of benefits generated, including reports of positive change across the dataset. For example, measures of cognition could be used to further explore positive change in keeping with participants’ reports of increased awareness and attention. This would allow researchers to gain a better understanding of the potential for quantifiable long-term gains to be generated as a result of engagement within water-based activities, including group-based surf therapy interventions described herein.

Within the present findings reside opportunities for transferability that may be applied in other clinical settings, in that these interventions need not be in the form of surfing specifically, an activity which may be somewhat restricted according to the appropriateness of a geographical location for completing water-based activities. There is a growing body of evidence for the benefits of the use of water-based activities for the promotion of human wellbeing more broadly (see [53]), with a recent systematic review of 33 studies concluding that engagement in a variety of purposefully designed and structured water-based activities can generate improvements in mental health and psychosocial wellbeing relevant to individuals with mixed cognitive and physical disabilities (see [94]). As such, there is much evidence to suggest that blue space activities may improve wellbeing generally, particularly given that blue space interventions typically focus on engaging individuals in meaningful and active experiences in the natural environment [53, 94]. Theoretically, it is feasible to assume that other water-based interventions may cultivate wellbeing in participants, providing that they also facilitate the experiences and associated mechanisms reported upon in our study (i.e. providing opportunities for acceptance of difficult emotions, community, achievement, meaning and purpose, physical exercise etc) and/or psychological processes noted within the wider literature (i.e. feelings of empowerment and respite—see [55] for context). In this study, surfing was chosen as a vehicle to connect participants to blue spaces and positive experiences, in addition to other determinants of wellbeing (i.e., positive health behaviours, connection to others and the self etc). The evidence-base surrounding surf therapy in particular is far more developed than that for other water-based activities, although it is possible that other water-based activities (such as canoeing, for example) could tap into the same key determinants and elicit similar benefits. How specific our findings are to the activity of surfing itself is unclear, highlighting the need for more research in this area. Randomised controlled trials could compare surf therapy with other blue space interventions, and the inclusion of appropriate control groups (i.e., outdoor vs indoor water-based settings) may help to elucidate whether there are any features unique to surfing that create the conditions for wellbeing, over and above other water-based activities, when delivered in a natural versus an artificial setting. However, a key feature of our study (regardless of the actual activity) is that it increased the accessibility of physical exercise and connection with nature. This is important given that many of the participants in the present study and in this population struggle to access blue spaces, highlighting barriers and opportunities that must be considered prior to using nature as therapy.

### Conclusions

The themes generated in the present study draw upon latest advancements in wellbeing science to describe how positive change may be facilitated post-ABI. The dynamic interactions between the different themes further highlight the importance of providing holistic care. We argue that immersing ABI survivors in their natural and social ecologies can facilitate a cascade of mechanisms for positive change (Fig 1). These findings and the interconnectedness of determinants of wellbeing emphasise a need to consider the individual in relation to their communities and natural environments, taking into consideration socio-contextual factors and associated barriers to (and opportunities for) neurorehabilitation. Using recent developments in wellbeing science to understand participant experiences of a novel nature-based intervention, the present study offers insights that extend on the Holistic Model of Neurorehabilitation, highlighting how community partnerships may be leveraged to create opportunities for wellbeing in those living with ABI.

## Supporting information

S1 File (DOCX)

## References

[1] Barber S., et al., House of Commons Library, Debate Pack: Acquired Brain Injury. 2018.

[2] Milders M., Fuchs S., and Crawford J.R., Neuropsychological Impairments and Changes in Emotional and Social Behaviour Following Severe Traumatic Brain Injury. Journal of Clinical and Experimental Neuropsychology, 2003. 25(2): p. 157–172. doi: 10.1076/jcen.25.2.157.13642 12754675

[3] Glenn Mel, et al., Depression amongst outpatients with traumatic brain injury. Brain Injury, 2001. 15(9): p. 811–818. doi: 10.1080/02699050010025777 11516349

[4] Jorge R.E., et al., Major Depression Following Traumatic Brain Injury. Archives of General Psychiatry, 2004. 61(1): p. 42–50. doi: 10.1001/archpsyc.61.1.42 14706943

[5] Osborn A.J., Mathias J.L., and Fairweather-Schmidt A.K., Depression following adult, non-penetrating traumatic brain injury: A meta-analysis examining methodological variables and sample characteristics. Neuroscience & Biobehavioral Reviews, 2014. 47: p. 1–15. doi: 10.1016/j.neubiorev.2014.07.007 25038422

[6] Anderson R.M., Patient empowerment and the traditional medical model. A case of irreconcilable differences? Diabetes Care, 1995. 18(3): p. 412–5. doi: 10.2337/diacare.18.3.412 7555490

[7] Hoofien D., et al., Traumatic brain injury (TBI) 10–20 years later: a comprehensive outcome study of psychiatric symptomatology, cognitive abilities and psychosocial functioning. Brain Inj, 2001. 15(3): p. 189–209. doi: 10.1080/026990501300005659 11260769

[8] Olver J.H., Ponsford J.L., and Curran C.A., Outcome following traumatic brain injury: a comparison between 2 and 5 years after injury. Brain Inj, 1996. 10(11): p. 841–8. doi: 10.1080/026990596123945 8905161

[9] Lefebvre H., Cloutier G., and Josée Levert M., Perspectives of survivors of traumatic brain injury and their caregivers on long-term social integration. Brain Inj, 2008. 22(7–8): p. 535–43. doi: 10.1080/02699050802158243 18568706

[10] Colantonio A., et al., Long-term outcomes after moderate to severe traumatic brain injury. Disabil Rehabil, 2004. 26(5): p. 253–61. doi: 10.1080/09638280310001639722 15200240

[11] Forslund M.V., et al., Global Outcome Trajectories up to 10 Years After Moderate to Severe Traumatic Brain Injury. Front Neurol, 2019. 10: p. 219. doi: 10.3389/fneur.2019.00219 30923511 PMC6426767

[12] Ponsford J.L., et al., Longitudinal follow-up of patients with traumatic brain injury: outcome at two, five, and ten years post-injury. J Neurotrauma, 2014. 31(1): p. 64–77. doi: 10.1089/neu.2013.2997 23889321

[13] Wong P.T.P., Second wave positive psychology’s (PP 2.0) contribution to counselling psychology. Counselling Psychology Quarterly, 2019. 32(3–4): p. 275–284.

[14] Wong P.T.P., Mayer C.-H., and Arslan G., Editorial: COVID-19 and Existential Positive Psychology (PP2.0): The New Science of Self-Transcendence. Frontiers in psychology, 2021. 12: p. 800308–800308. doi: 10.3389/fpsyg.2021.800308 34956025 PMC8699172

[15] Ben-Yishay Y., Reflections on the Evolution of the Therapeutic Milieu Concept. Neuropsychological Rehabilitation, 1996. 6(4): p. 327–343.

[16] Prigatano G.P., Principles of neuropsychological rehabilitation. 1999: Oxford University Press.

[17] Cattelani R., Zettin M., and Zoccolotti P., Rehabilitation treatments for adults with behavioral and psychosocial disorders following acquired brain injury: A systematic review. Neuropsychology review, 2010. 20(1): p. 52–85. doi: 10.1007/s11065-009-9125-y 20143264

[18] Cicerone K.D., et al., A randomized controlled trial of holistic neuropsychologic rehabilitation after traumatic brain injury. Arch Phys Med Rehabil, 2008. 89(12): p. 2239–49. doi: 10.1016/j.apmr.2008.06.017 19061735

[19] Ben-Yishay Y. and Diller L., Handbook of holistic neuropsychological rehabilitation: outpatient rehabilitation of traumatic brain injury. 2011: Oxford University Press. doi: 10.1101/gad.2041611

[20] Leonardi M. and Martinuzzi A., ICF and ICF-CY for an innovative holistic approach to persons with chronic conditions. Disabil Rehabil, 2009. 31 Suppl 1: p. S83–7. doi: 10.3109/09638280903317948 19968542

[21] Tate D.G. and Pledger C., An integrative conceptual framework of disability. New directions for research. Am Psychol, 2003. 58(4): p. 289–95. doi: 10.1037/0003-066x.58.4.289 12866395

[22] Wilkie L., et al., The Impact of Psycho-Social Interventions on the Wellbeing of Individuals With Acquired Brain Injury During the COVID-19 Pandemic. Frontiers in Psychology, 2021. 12(793). doi: 10.3389/fpsyg.2021.648286 33841287 PMC8027334

[23] Fisher Z., et al., Emotion, Wellbeing and the Neurological Disorders. 2020.

[24] Rabinowitz A.R. and Arnett P.A., Positive psychology perspective on traumatic brain injury recovery and rehabilitation. Appl Neuropsychol Adult, 2018. 25(4): p. 295–303. doi: 10.1080/23279095.2018.1458514 29781729

[25] Andrewes H.E., Walker V., and O’Neill B., Exploring the use of positive psychology interventions in brain injury survivors with challenging behaviour. Brain Injury, 2014. 28(7): p. 965–971. doi: 10.3109/02699052.2014.888764 24826958

[26] Cullen B., et al., Positive PsychoTherapy in ABI Rehab (PoPsTAR): A pilot randomised controlled trial. Neuropsychological Rehabilitation, 2018. 28(1): p. 17–33. doi: 10.1080/09602011.2015.1131722 26726854

[27] Tulip C., et al., Building Wellbeing in People With Chronic Conditions: A Qualitative Evaluation of an 8-Week Positive Psychotherapy Intervention for People Living With an Acquired Brain Injury. Frontiers in Psychology, 2020. 11(66). doi: 10.3389/fpsyg.2020.00066 32082221 PMC7006056

[28] Barak Y. and Achiron A., Happiness and neurological diseases. Expert Rev Neurother, 2009. 9(4): p. 445–59. doi: 10.1586/ern.09.1 19344298

[29] Carr A., et al., Effectiveness of positive psychology interventions: a systematic review and meta-analysis. The Journal of Positive Psychology, 2020: p. 1–21.

[30] Buecker S., et al., Physical activity and subjective well-being in healthy individuals: a meta-analytic review. Health Psychol Rev, 2020: p. 1–19. doi: 10.1080/17437199.2020.1719368 32452716

[31] Archer T., Influence of Physical Exercise on Traumatic Brain Injury Deficits: Scaffolding Effect. Neurotoxicity Research, 2012. 21(4): p. 418–434. doi: 10.1007/s12640-011-9297-0 22183422

[32] Haslam C., Jetten J., Cruwys T., Dingle G.A., & Haslam S.A, The New Psychology of Health: Unlocking the Social Cure. 1st ed. 2018: Routledge.

[33] Martin R., Levack W.M.M., and Sinnott K.A., Life goals and social identity in people with severe acquired brain injury: an interpretative phenomenological analysis. Disability and Rehabilitation, 2015. 37(14): p. 1234–1241. doi: 10.3109/09638288.2014.961653 25250809

[34] Capaldi C.A., et al., Flourishing in nature: A review of the benefits of connecting with nature and its application as a wellbeing intervention. International Journal of Wellbeing, 2015. 5(4).

[35] Vibholm A.P., Christensen J.R., and Pallesen H., Nature-based rehabilitation for adults with acquired brain injury: a scoping review. International Journal of Environmental Health Research, 2020. 30(6): p. 661–676. doi: 10.1080/09603123.2019.1620183 31131619

[36] Kwasnicka D., et al., Theoretical explanations for maintenance of behaviour change: a systematic review of behaviour theories. Health Psychology Review, 2016. 10(3): p. 277–296. doi: 10.1080/17437199.2016.1151372 26854092 PMC4975085

[37] Mead J., Fisher Z., and Kemp A., Moving Beyond Disciplinary Silos Towards a Transdisciplinary Model of Wellbeing: An Invited Review. Frontiers in Psychology, 2021. 12. 10.3389/fpsyg.2021.642093 PMC8160439 34054648

[38] Kemp A.H. and Fisher Z., Wellbeing, Whole Health and Societal Transformation: Theoretical Insights and Practical Applications. Global Advances in Health and Medicine, 2022. 11: p. 21649561211073077. doi: 10.1177/21649561211073077 35096491 PMC8796073

[39] Kemp A., Arias J., and Fisher Z., Social Ties, Health and Wellbeing: A Literature Review and Model. 2018.

[40] Haig B.D., Exploratory Factor Analysis, Theory Generation, and Scientific Method. Multivariate Behavioral Research, 2005. 40(3): p. 303–329. doi: 10.1207/s15327906mbr4003_2 26794686

[41] Kaplan R. and Kaplan S., The experience of nature: A psychological perspective. 1989: CUP Archive.

[42] Kaplan S., The restorative benefits of nature: Toward an integrative framework. Journal of Environmental Psychology, 1995. 15(3): p. 169–182.

[43] Bowler D.E., et al., A systematic review of evidence for the added benefits to health of exposure to natural environments. BMC Public Health, 2010. 10: p. 456. doi: 10.1186/1471-2458-10-456 20684754 PMC2924288

[44] Tsunetsugu Y., Park B.-J., and Miyazaki Y., Trends in research related to “Shinrin-yoku” (taking in the forest atmosphere or forest bathing) in Japan. Environmental Health and Preventive Medicine, 2009. 15(1): p. 27. 10.1007/s12199-009-0091-z PMC2793347 19585091

[45] Hansmann R., Hug S.-M., and Seeland K., Restoration and stress relief through physical activities in forests and parks. Urban Forestry & Urban Greening, 2007. 6: p. 213–225.

[46] Ulrich R.S., Natural Versus Urban Scenes: Some Psychophysiological Effects. Environment and Behavior, 1981. 13(5): p. 523–556.

[47] Ulrich R.S., et al., Stress recovery during exposure to natural and urban environments. Journal of environmental psychology, 1991. 11(3): p. 201–230.

[48] Lukow H.R.I., et al., Relationship Between Resilience, Adjustment, and Psychological Functioning After Traumatic Brain Injury: A Preliminary Report. The Journal of Head Trauma Rehabilitation, 2015. 30(4): p. 241–248. doi: 10.1097/HTR.0000000000000137 25931185

[49] Meredith G.R., et al., Minimum Time Dose in Nature to Positively Impact the Mental Health of College-Aged Students, and How to Measure It: A Scoping Review. Frontiers in Psychology, 2020. 10(2942). 10.3389/fpsyg.2019.02942 PMC6970969 31993007

[50] Berman M.G., Jonides J., and Kaplan S., The Cognitive Benefits of Interacting With Nature. Psychological Science, 2008. 19(12): p. 1207–1212. doi: 10.1111/j.1467-9280.2008.02225.x 19121124

[51] Berto R., Exposure to restorative environments helps restore attentional capacity. Journal of Environmental Psychology, 2005. 25(3): p. 249–259.

[52] Csikszentmihalyi M., Abuhamdeh S., and Nakamura J., Flow, in Flow and the foundations of positive psychology. 2014, Springer. p. 227–238.

[53] Foley R. and Kistemann T., Blue space geographies: Enabling health in place. Health Place, 2015. 35: p. 157–65. doi: 10.1016/j.healthplace.2015.07.003 26238330

[54] Gascon M., et al., Outdoor blue spaces, human health and well-being: A systematic review of quantitative studies. Int J Hyg Environ Health, 2017. 220(8): p. 1207–1221. doi: 10.1016/j.ijheh.2017.08.004 28843736

[55] Marshall J., et al., “When I was surfing with those guys I was surfing with family.” A Grounded Exploration of Program Theory within the Jimmy Miller Memorial Foundation Surf Therapy Intervention. Global Journal of Community Psychology Practice, 2020. 11(2).

[56] Mead J., et al., Rethinking wellbeing: Toward a more ethical science of wellbeing that considers current and future generations. 2019.

[57] Rzezak P., et al., Affective responses after different intensities of exercise in patients with traumatic brain injury. Frontiers in psychology, 2015. 6: p. 839. doi: 10.3389/fpsyg.2015.00839 26161074 PMC4479709

[58] Van Cappellen P., et al., Positive affective processes underlie positive health behaviour change. Psychology & Health, 2018. 33(1): p. 77–97. doi: 10.1080/08870446.2017.1320798 28498722 PMC5682236

[59] Thompson Coon J., et al., Does participating in physical activity in outdoor natural environments have a greater effect on physical and mental wellbeing than physical activity indoors? A systematic review. Environ Sci Technol, 2011. 45(5): p. 1761–72. doi: 10.1021/es102947t 21291246

[60] Pasanen T.P., Tyrväinen L., and Korpela K.M., The Relationship between Perceived Health and Physical Activity Indoors, Outdoors in Built Environments, and Outdoors in Nature. Applied Psychology: Health and Well-Being, 2014. 6(3): p. 324–346. 25044598 10.1111/aphw.12031 PMC4233975

[61] White M.P., et al., Spending at least 120 minutes a week in nature is associated with good health and wellbeing. Scientific Reports, 2019. 9(1): p. 7730. doi: 10.1038/s41598-019-44097-3 31197192 PMC6565732

[62] Braun V. and Clarke V., Using thematic analysis in psychology. Qualitative Research in Psychology, 2006. 3(2): p. 77–101.

[63] Braun V. and Clarke V., Successful Qualitative Research: A Practical Guide for Beginners. 2013.

[64] Archer M., et al., Critical realism: Essential readings. 2013: Routledge.

[65] Capaldi C.A., Dopko R.L., and Zelenski J.M., The relationship between nature connectedness and happiness: a meta-analysis. Frontiers in Psychology, 2014. 5(976). doi: 10.3389/fpsyg.2014.00976 25249992 PMC4157607

[66] Porges S.W., The polyvagal theory: neurophysiological foundations of emotions, attachment, communication, and self-regulation (Norton Series on Interpersonal Neurobiology). 2011: WW Norton & Company.

[67] Porges S.W., Making the world safe for our children: Down-regulating defence and up-regulating social engagement to’optimise’the human experience. Children Australia, 2015. 40(2): p. 114.

[68] Kemp A.H. and Quintana D.S., The relationship between mental and physical health: Insights from the study of heart rate variability. International Journal of Psychophysiology, 2013. 89(3): p. 288–296. doi: 10.1016/j.ijpsycho.2013.06.018 23797149

[69] Walter K.H., et al., Surf Therapy Practice, Research, and Coalition Building: Future Directions. Global Journal of Community Psychology Practice, 2020. 11(2).

[70] Caddick N., Smith B., and Phoenix C., The Effects of Surfing and the Natural Environment on the Well-Being of Combat Veterans. Qualitative Health Research, 2015. 25(1): p. 76–86. doi: 10.1177/1049732314549477 25189537

[71] Baer R., Smith G., and Allen K., Assessment of Mindfulness by Self-Report: The Kentucky Inventory of Mindfulness Skills. Assessment, 2004. 11: p. 191–206. doi: 10.1177/1073191104268029 15358875

[72] Cardaciotto L., et al., The assessment of present-moment awareness and acceptance: the Philadelphia Mindfulness Scale. Assessment, 2008. 15(2): p. 204–23. doi: 10.1177/1073191107311467 18187399

[73] Kohls N., Sauer S., and Walach H., Facets of mindfulness–Results of an online study investigating the Freiburg mindfulness inventory. Personality and Individual Differences, 2009. 46: p. 224–230.

[74] Ford B.Q., et al., The psychological health benefits of accepting negative emotions and thoughts: Laboratory, diary, and longitudinal evidence. Journal of Personality and Social Psychology, 2018. 115(6): p. 1075–1092. doi: 10.1037/pspp0000157 28703602 PMC5767148

[75] Weinstein A.A., et al., Effect of Aerobic Exercise Training on Mood in People With Traumatic Brain Injury: A Pilot Study. The Journal of Head Trauma Rehabilitation, 2017. 32(3). doi: 10.1097/HTR.0000000000000253 PMC5339064 27603762

[76] Ryff C.D., Happiness is everything, or is it? Explorations on the meaning of psychological well-being. Journal of personality and social psychology, 1989. 57(6): p. 1069–1081.

[77] Seligman M.E.P., Flourish: a new understanding of happiness and well-being—and how to achieve them / Martin E.P. Seligman. 2011: London: Nicholas Brealey Pub.

[78] Schueller S.M. and Seligman M.E.P., Pursuit of pleasure, engagement, and meaning: Relationships to subjective and objective measures of well-being. The Journal of Positive Psychology, 2010. 5(4): p. 253–263.

[79] Birgit, et al., Prognostic factors of return to work after traumatic or non-traumatic acquired brain injury. Disability and Rehabilitation, 2016. 38(8): p. 733–741. doi: 10.3109/09638288.2015.1061608 26138021

[80] van Velzen J.M., et al., Prognostic factors of return to work after acquired brain injury: A systematic review. Brain Injury, 2009. 23(5): p. 385–395. doi: 10.1080/02699050902838165 19408163

[81] Fredrickson B.L., Chapter One—Positive Emotions Broaden and Build, in Advances in Experimental Social Psychology, Devine P.and Plant A., Editors. 2013, Academic Press. p. 1–53.

[82] Britton E., Kindermann G., and Carlin C., Surfing and the Senses: Using Body Mapping to Understand the Embodied and Therapeutic Experiences of Young Surfers with Autism. 2020.

[83] Dennis C.-L., Peer support within a health care context: a concept analysis. International Journal of Nursing Studies, 2003. 40(3): p. 321–332. doi: 10.1016/s0020-7489(02)00092-5 12605954

[84] Bandura A., Social learning theory. 1977.

[85] Salas C.E., et al., “Relating through sameness”: a qualitative study of friendship and social isolation in chronic traumatic brain injury. Neuropsychological Rehabilitation, 2018. 28(7): p. 1161–1178. doi: 10.1080/09602011.2016.1247730 27802787

[86] Godfrey C., Devine-Wright H., and Taylor J., The positive impact of structured surfing courses on the wellbeing of vulnerable young people. Community Practitioner, 2015. 88. 26357740

[87] Gaspar de Matos M., et al., Surfing for Social Integration: Mental Health and Well-Being promotion through Surf Therapy among Institutionalized Young People. HSOA Journal of Community Medicine and Public Health Care, 2017. 4: p. 026.

[88] Ownsworth T., Self-identity after brain injury. 2014: Psychology Press.

[89] Cohen S. Social Relationships and Health. American Psychological Association doi: 10.1037/0003-066X.59.8.676 2004. 15554821

[90] Levy B.B., et al., Peer support interventions for individuals with acquired brain injury, cerebral palsy, and spina bifida: a systematic review. BMC Health Services Research, 2019. 19(1): p. 288. doi: 10.1186/s12913-019-4110-5 31068184 PMC6505073

[91] Hughes R., Fleming P., and Henshall L., Peer support groups after acquired brain injury: a systematic review. Brain Injury, 2020. 34(7): p. 847–856. doi: 10.1080/02699052.2020.1762002 32421382

[92] Cicerone K.D. and Azulay J., Perceived self-efficacy and life satisfaction after traumatic brain injury. The Journal of Head Trauma Rehabilitation, 2007. 22(5): p. 257–266. doi: 10.1097/01.HTR.0000290970.56130.81 17878767

[93] Kiken L.G., et al., From a state to a trait: Trajectories of state mindfulness in meditation during intervention predict changes in trait mindfulness. Personality and individual differences, 2015. 81: p. 41–46. doi: 10.1016/j.paid.2014.12.044 25914434 PMC4404745

[94] Britton E., et al., Blue care: a systematic review of blue space interventions for health and wellbeing. Health promotion international, 2020. 35(1): p. 50–69. doi: 10.1093/heapro/day103 30561661 PMC7245048

[95] Hignett A., et al., Evaluation of a surfing programme designed to increase personal well-being and connectedness to the natural environment among ‘at risk’ young people. Journal of Adventure Education and Outdoor Learning, 2018. 18(1): p. 53–69.

[96] Fleischmann D., et al., Surf medicine: Surfing as a means of therapy for combat-related polytrauma. JPO: Journal of Prosthetics and Orthotics, 2011. 23(1): p. 27–29.

[97] Hamel R.N. and Smoliga J.M., Physical Activity Intolerance and Cardiorespiratory Dysfunction in Patients with Moderate-to-Severe Traumatic Brain Injury. Sports Medicine, 2019. 49(8): p. 1183–1198. doi: 10.1007/s40279-019-01122-9 31098990

[98] Halvorsrud K., et al., Identifying evidence of effectiveness in the co-creation of research: a systematic review and meta-analysis of the international healthcare literature. Journal of Public Health, 2019. 43(1): p. 197–208. 10.1093/pubmed/fdz126 PMC8042368 31608396

[99] de Bell S., et al., The importance of nature in mediating social and psychological benefits associated with visits to freshwater blue space. Landscape and Urban Planning, 2017. 167: p. 118–127.

[100] Uchida Y. and Oishi S., The Happiness of Individuals and the Collective. Japanese Psychological Research, 2016. 58(1): p. 125–141.

[101] Martin L., et al., Nature contact, nature connectedness and associations with health, wellbeing and pro-environmental behaviours. Journal of Environmental Psychology, 2020. 68: p. 101389.

[102] Pritchard A., et al., The Relationship Between Nature Connectedness and Eudaimonic Well-Being: A Meta-analysis. Journal of Happiness Studies, 2020. 21.

[103] Arem H., et al., Leisure time physical activity and mortality: a detailed pooled analysis of the dose-response relationship. JAMA internal medicine, 2015. 175(6): p. 959–967. doi: 10.1001/jamainternmed.2015.0533 25844730 PMC4451435

[104] Chekroud S., et al., Association between physical exercise and mental health in 1·2 million individuals in the USA between 2011 and 2015: a cross-sectional study. The Lancet Psychiatry, 2018. 5. 10.1016/S2215-0366(18)30227-X 30099000

