# Supplementary material for: Riding the wave into wellbeing: A qualitative evaluation of surf therapy for individuals living with acquired brain injury
Source: PLoS One. 2022 Apr 7;17(4):e0266388. doi: 10.1371/journal.pone.0266388 (PMC8989185; doi:10.1371/journal.pone.0266388)
Supplement: S1 File — (DOCX) [file pone.0266388.s001.docx]

Surfability Project

**Semi-structured Topic Guide**

1. Tell me about how your life has changed following your brain injury?

2. Tell me about how the brain injury has affected your approach to new situations or activities?

3. When you first heard about Surfability, what was your initial reaction?

4. What did you hope to get from attending Surfability? Why was this important to you?

5. What was your experience of being involved with Surfability?

6. What did it feel like when you got in the water for the first time? (- got on the surfboard?)

7. Tell me about how you felt after each session? Did you notice feeling anything different in yourself or towards others, or the world/environment?

8. Has being part of this group changed how you feel or think about yourself after your brain injury? If so in what ways?

9. Some people living with brain injury report feeling socially isolated. What are your thoughts about that, and do you feel Surfability changed that at all?

10. Some people living with brain injury report feeling restricted in terms of their ability to engage in sport and exercise. What are your thoughts on this? Has attending Surfability changed this in any way?

11. Some people living with brain injury say that they experience low mood or anxiety. What are your thoughts about this? Has anything changed since attending Surfability?

12. Is there anything you would like to change from your experience of this group?

- Time

- Length of sessions

- Day

- Group format?

- Changing facilities?

- Access difficulties?

13. What would you say to someone else if they were in a similar situation and were given the opportunity to go surfing?

14. Has anything changed for you since attending Surfability?

15. Is there anything you would like to add, or anything you think I should know to fully understand your experiences of the group?
